# Complete genome sequence analysis of human echovirus 30 isolated during a large outbreak in Guangdong Province of China, in 2012

**DOI:** 10.1007/s00705-013-1818-0

**Published:** 2013-08-29

**Authors:** Hong Xiao, Keyong Huang, Ling Li, Xianbo Wu, Li Zheng, Chengsong Wan, Wei Zhao, Changwen Ke, Bao Zhang

**Affiliations:** 1 Lab of Biosafety Level 3, Southern Medical University, Guangzhou, 510515 People’s Republic of China; 2 Institute of Microbiology, Center for Diseases Control and Prevention of Guangdong Province, 176 Xin Gang West Road, Guangzhou, Guangdong 510300 People’s Republic of China

## Abstract

In May and June 2012, an outbreak of aseptic meningitis caused by Echovirus 30 (E30) occurred on a large scale in Luoding, Guangdong Province, China. Our team successfully isolated one subtype, strain 2012EM161, and its complete genome was sequenced. The phylogenetic tree of viral protein (VP) 1 gene sequences showed that the viral isolate was similar to the E30 strain prevalent in Fujian (2011), with identity of 98.05–99.32 % and 98.63–99.32 % for nucleotides and amino acids respectively. Whole genome-based phylogenetic analysis indicated that 2012EM161 contained the most proximate consensus to DQ246620 (Zhejiang, 2003) and FDJS03 (AY948442, Jiangsu, 2005), with nucleotide homogeneity of 87.09 % and 86.98 % respectively. The RDP4.16 and Simplot analysis showed that the newly discovered 2012EM161 was probably a recombinant, which was closely related to the strain of E30 (DQ246620) in the first half of the genome and the strain of E6 (JX976771) in genomic P3 region. The whole genome sequence of 2012EM161 will allow further study of the origin, evolution, and the molecular epidemiology of E30 strains.

**Electronic supplementary material:**

The online version of this article (doi:10.1007/s00705-013-1818-0) contains supplementary material, which is available to authorized users.

## Introduction

Echovirus 30 (E30), with an ssRNA positive-stranded genome, is a member of the genus Enterovirus in the family Picornaviridae and belongs to human enterovirus subgroup B (HEV-B) [7]. It is a major pathogen associated with aseptic meningitis, which has occurred at high prevalence in recent decades all over the world, including America [13], Canada [4], France [12], Italy [5], Germany [15], England [2], Japan [8], Korea [9], India [11], Taiwan [3, 10], and the Chinese Mainland. In China, several E30 epidemics have been recorded, in Jiangsu [18], Shandong [16], Fujian [17] and other provinces. In 2012, an epidemic of E30 virus infection that involved 183 children occurred in Luoding, Guangdong province, and this was the first E30 epidemic reported in Guangdong Province. We successfully isolated a virus strain from one of the patients which was identified as E30 by complete genome sequencing.

## Provenance of the virus materials

The strain 2012EM161 was isolated from the cerebrospinal fluid (CSF) of a female infant who suffered aseptic meningitis during the epidemic in Luoding, Guangdong Province. This female infant recovered after one week of symptomatic treatment. The isolated strain was identified as E30 by immunological detection, fluorescence quantitative PCR and sequencing of the VP1 region.

Human rhabdomyosarcoma (RD) cell line was used for expansion and passage of the 2012EM161 strain. RD cell line firstly established by McAllister et al. [14] is useful for the diagnosis of most of the important enterovirus infections [1]. Cells were cultured in DMEM (Dulbecco’s Modified Eagle’s Medium) containing 10 % fetal bovine serum (FBS) in a humidified incubator at 37 °C with 5 % CO_2_. The RNA was extracted using a viral extraction kit (Qiagen), and its 3’ end was amplified and sequenced using a 3’-full RACE core set (Takara, Cat:6106). Primers were designed according to conserved sequences in the whole genome sequences of fourteen strains of E30 reported in Genbank and were used to determine the 5’ and internal viral sequences using an RNA PCR kit ver3.0 (TaKaRa, Cat:Drr019A). The primers used are listed in Supplementary Table 1.

Sanger sequencing was applied on an AB3730 DNA analyzer, and sequence assembly was performed with DNASTAR7.0 software. The tree was constructed with MEGA 5.1 software using the neighbor-joining method with 1,000 bootstrap replicates. The VP1 sequence and whole genome sequence are listed in Supplementary Table 2 and Table 3 respectively. RDP4.16 and simplot3.5.1 were used to analyze the recombinant sequences of several strains of HEV-B (Supplementary Table 3).

## Sequence properties

**Fig. 1:**
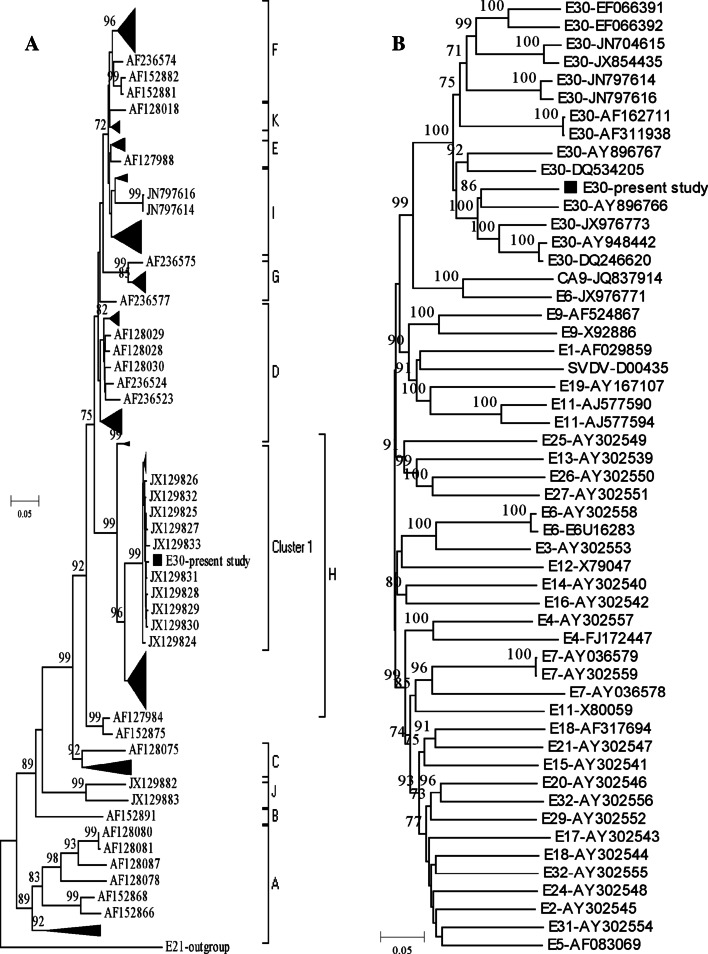
Phylogenetic trees construction with VP1 protein and complete genome of Echovirus E30. **A** VP1 protein with 876 bp of 246 strains Echovirus E30; **B** genome sequence (nt94-7367) of some Human entervirus B protype and SVDV genome for reference. The nucleotide position is based on the E30 of 2012EM161 stain. The trees were built with NJ method using MEGA 5.1 software and tested with 1000 bootstrap replicates

The full-length genome of 2012EM161 comprised 7427 bp and included a 741 bp 5′ UTR, a 101 bp 3′ UTR and an open reading frame encoding a 2194 amino acid polyprotein, the cleavage site of which was consistent with JX854435 [6]. The phylogenetic tree based on the VP1 region showed that 2012EM161 was located in the cluster 1 of h lineage of E30 (Fig. 1A), with similarity values of 98.63 % to 99.32 %. The cluster 1, including the isolates from JX129810 to JX129833, together other isolates caused 284 cases diagnosed with aseptic meningitis in Fujian Province of China from April to June in 2011 [17]. In addition, the VP1 gene’s nucleotide/amino acid homology values of the 2012EM161 strain with DQ246620 and FDJS03 (Genbank NO. AY948442) were 98.23 %/98.28 % and 94.29 %/98.29 % respectively. Strain of DQ246620 caused outbreak of aseptic meningitis in Zhejiang Province of China in 2003, while FDJS03 strain emerged outbreak in Jiangsu Province of China [19]. The above results proved that our isolated strain, 2012EM161, belonged to E30 type.

**Fig. 2:**
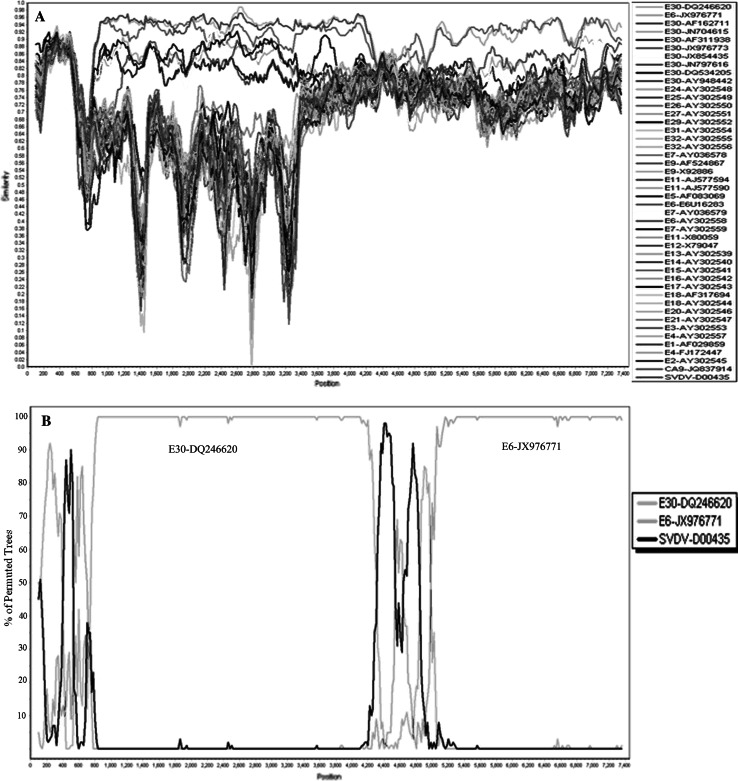
Similarity and bootscanning analysis of 2012 EM161 Echovirus E30 strain. **A** similarity plot analysis with other human entervirus B strains and SVDV for reference. **B** bootscanning result on the putative recombinant 2012EM161 strain and its parental sequences. The parameters are used as window size 200 bp, 20 bp step size, distance model of kirmura 2-parameter, NJ tree model, 100 pseudoreplicates. The arbitary recombinant threshold is 70 %

Complete genomic sequence (CGS) alignment in Genbank revealed that 2012EM161 had a high similarity to E30 strains from provinces of Zhejiang (DQ246620) and Jiangsu (AY948442), with the identity of 87.09 % and 86.98 % respectively. Phylogenetic analysis further demonstrated that 2012EM161 and the E30 strain of DQ246620 and AY948442 belonged to the same series (Fig. 1B). However, the score of individual VP1 gene alignment was higher than that of CGS, 98.23 %/98.28 % vs 87.09 %/86.98 % in DQ246620 and AY948442, which indicated that recombination had probably occurred in the 2012EM16 strain. The results of the RDP4.16 and simplot3.5.1 analysis showed that 2012EM161 had similarity of 75 %–85 % at the 5’ end and non-structural protein region within HEV B strains (Fig. 2A). Based on the current nucleotide dataset, Bootscan program analysis suggested that the isolated strain was a mosaic reassortant, which manifested the highest similarity to the strain of E30 from Zhejiang (DQ246620) in the 5’ half of the genome and the strain of E6 strain (JX976771) in the P3 region, with the potential initial recombination site at 4907 bp (Fig. 2B).”

Given that a whole genome sequence for the E30 strain responsible for the meningitis epidemic in Fujian (2011) [17] is unavailable, it is difficult to evaluate its homogeneity with the 2012EM161 strain of Guangdong (2012), or to determine whether 2012EM161 originated from Fujian. This will only be clarified when the whole genome sequence of the Fujian strain is obtained. This is the first report of the whole genome sequence of the E30 strain prevalent in Luoding (May to June, 2012), and it will contribute to further study of the molecular epidemiology, source, and evolution of E30 strains.

The complete genomic sequence of E30 2012EM161 strain was deposited in the Genbank database under accession no. KC897073.

## Electronic supplementary material

Below is the link to the electronic supplementary material.

Supplementary material 1 (PDF 11 kb)
