# Supplementary material for: Complete genome sequence analysis of human echovirus 30 isolated during a large outbreak in Guangdong Province of China, in 2012
Source: Arch Virol. 2013 Aug 29;159(2):379–83. doi: 10.1007/s00705-013-1818-0 (PMC3906529; doi:10.1007/s00705-013-1818-0)
Supplement: Supplementary file 1 — Supplementary material 1 (PDF 11 kb) [file 705_2013_1818_MOESM1_ESM.pdf]

Supplements Table 1

Table 1 Specific primers for PCR of 2012EM161 Echovirus 30 strain

primers	sequence	position
1F	TTAAAACAGCCTGTGGGTT	1-19
1R1	TTCAATTGTCACCATAAGCAG	584-604
1R	GATCTTGAGTGAAATCTTGCC	869-889
2F	AGCGGAACCGACTACTTT	534-551
2R	GCCTGCGTTATGGACTGC	1453-1470
3F	CGGTCAAGCAATGGAGTT	1395-1412
3R	GTAGCCCGCATCAGTGTA	2281-2298
4F	ACGTTGGCTTACAATCCAGC	2201-2220
4R	CCGAGACTCAGTCACTCCTTG	3253-3273
5F	ACAGCGTTGCTCGCGTCTA	3146-3164
5R	GCGTTGGTCATCTCCGTGAA	4090-4109
6F	TACTACGGAATCCCCATGGC	4039-4058
6R	TCTGTGGTTGTACTCCCTGAACAT	4996-5019
7F	ACATTGAAGTTATCTCCATGTACAG	4808-4832
7R	CGGCTTCCATCATGTACTCATC	6148-6169
8F1	AGTGTGGTGGTGTCCTGATGTC	5825-5846
8F2	GAACCAGCGGTGCTAAGGAACG	6052-6073
9F	AACTACATAGATTACTTATGCAACTC	6727-6752

Supplementary Table 2

Table 2. the accession number of VP1 sequences for the phylogenetic tree construction

E21-AY302547	AF128080	AF236574	AY371473	FJ868361	JX129832
E30-present study	AF128081	AF236575	AY371479	FJ919565	JX129833
AF081340	AF128087	AF236577	AY371481	GQ329836	JX129834
AF081341	AF128090	AF236578	AY665608	GQ329838	JX129835
AF127984	AF152866	AF311938	AY695092	GQ352377	JX129836
AF127988	AF152868	AJ133658	AY695094	GU142904	JX129837
AF127989	AF152871	AJ133659	AY695095	HQ436346	JX129838
AF127991	AF152875	AJ133660	AY695101	HQ436347	JX129839
AF127994	AF152876	AJ241448	AY695102	HQ625643	JX129840
AF127995	AF152879	AJ241453	AY695103	HQ625644	JX129841
AF127999	AF152880	AJ241456	AY695105	HQ625649	JX129842
AF128001	AF152881	AJ276813	AY695106	JN177727	JX129843
AF128003	AF152882	AJ276815	AY695107	JN203946	JX129844
AF128006	AF152884	AJ430699	AY879317	JN203948	JX129845
AF128008	AF152886	AJ430700	AY879319	JN203949	JX129846
AF128011	AF152887	AJ430701	AY879322	JN203957	JX129847
AF128018	AF152888	AJ430703	AY879324	JN704615	JX129848
AF128020	AF152891	AJ430704	AY879326	JN797614	JX129849
AF128021	AF162711	AJ430705	AY948442	JN797616	JX129850
AF128023	AF236517	AM236607	DQ118682	JX129810	JX129851
AF128025	AF236518	AM236610	DQ118694	JX129811	JX129852
AF128027	AF236519	AM236612	DQ246620	JX129812	JX129853
AF128028	AF236520	AM236619	DQ534205	JX129813	JX129854
AF128029	AF236523	AM236989	DQ842246	JX129814	JX129861
AF128030	AF236524	AM236990	DQ842249	JX129815	JX129863
AF128031	AF236531	AM710997	DQ842256	JX129816	JX129866
AF128032	AF236532	AM711007	DQ842261	JX129817	JX129870
AF128036	AF236536	AM711027	DQ842263	JX129818	JX129873
AF128037	AF236540	AM711030	DQ842264	JX129819	JX129874
AF128039	AF236542	AM946155	EF066391	JX129820	JX129875
AF128040	AF236548	AM946168	EF066392	JX129821	JX129876
AF128042	AF236549	AM946175	EF397655	JX129822	JX129877
AF128055	AF236550	AY146073	EF397656	JX129823	JX129878
AF128061	AF236552	AY146076	EU004586	JX129824	JX129879
AF128063	AF236556	AY146080	EU004588	JX129825	JX129880
AF128064	AF236557	AY146081	EU280291	JX129826	JX129881
AF128068	AF236560	AY146082	EU280298	JX129827	JX129882
AF128069	AF236562	AY146084	EU293769	JX129828	JX129883
AF128072	AF236563	AY371457	EU293774	JX129829	JX513544
AF128075	AF236565	AY371460	EU293777	JX129830	JX854435
AF128078	AF236573	AY371462	EU293778	JX129831	JX976773

Supplement table 3.

Table 3. the accession number of genome sequences for the phylogenetic tree construction

E30-present study	E30-AY948442
CA9-JQ837914	E30-DQ246620
E11-AJ577590	E30-DQ534205
E11-AJ577594	E30-EF066391
E11-X80059	E30-EF066392
E12-X79047	E30-JN704615
E13-AY302539	E30-JN797614
E14-AY302540	E30-JN797616
E15-AY302541	E30-JX854435
E16-AY302542	E30-JX976773
E17-AY302543	E31-AY302554
E18-AF317694	E32-AY302555
E18-AY302544	E32-AY302556
E19-AY167107	E3-AY302553
E1-AF029859	E4-AY302557
E20-AY302546	E4-FJ172447
E21-AY302547	E5-AF083069
E24-AY302548	E6-AY302558
E25-AY302549	E6-E6U16283
E26-AY302550	E6-JX976771
E27-AY302551	E7-AY036578
E29-AY302552	E7-AY036579
E2-AY302545	E7-AY302559
E30-AF162711	E9-AF524867
E30-AF311938	E9-X92886
E30-AY896766	SVDV-D00435
E30-AY896767	
